# Prenatal Diagnosis and Pregnancy Outcomes in Fetuses With Increased Nuchal Translucency Thickness

**DOI:** 10.1155/jp/6457722

**Published:** 2026-09-03

**Authors:** Yanchun Zhang, Hongyan Xu, Lijing Wang, Wen Zhang, Kaibo Liu

**Affiliations:** ^1^ Department of Perinatal Health, Beijing Obstetrics and Gynecology Hospital, Capital Medical University, Beijing, China, ccmu.edu.cn; ^2^ Department of Perinatal Health, Beijing Maternal and Child Health Care Hospital, Beijing, China

**Keywords:** chromosome abnormality, congenital heart disease, intervention prenatal diagnosis, nuchal translucency thickness, pregnancy outcomes, ultrasound prenatal diagnosis

## Abstract

**Objective:**

To evaluate prenatal diagnosis and pregnancy outcomes in fetuses with nuchal translucency (NT) thickening ≥ 95^th^ percentile (P_95_).

**Methods:**

In total, 3328 singleton pregnancies diagnosed with increased NT thickness on first‐trimester ultrasound screening were retrospectively included. In addition, second‐trimester ultrasound, noninvasive prenatal testing, and follow‐up data were collected.

**Results:**

There were 2815 patients with isolated fetal NT thickening (Group A) and 513 patients with fetal NT thickening combined with other abnormalities (Group B). The prenatal ultrasound diagnosis rate was 44.29%, and the prenatal ultrasound diagnosis concordance rate in Group B was greater than that in Group A (*p* < 0.001). The chromosomal abnormality rates in Groups A and B were 20.00% and 51.22%, respectively. Moreover, the incidence of chromosomal abnormalities in Group B was greater than that in Group A, and it increased with increasing NT thickness in Groups A and B (both *p* < 0.001). The most common chromosomal abnormality was Trisomy 21. In addition, the structural abnormalities and soft indicators observed on ultrasound included congenital heart disease and cystic hygroma. The live birth rates were 82.31% in Group A and 13.84% in Group B (*p* < 0.001). Furthermore, after the termination group was excluded, with increasing NT thickness, the live birth rate decreased, whereas the spontaneous abortion/embryo arrest rate increased (both *p* < 0.001). With respect to live births, NT thickness was negatively correlated (*p* < 0.05) in Groups A and B, and maternal age was positively correlated in Group A (*p* < 0.001).

**Conclusions:**

NT thickening suggests a greater risk of chromosomal abnormalities (especially Trisomy 21), and this risk is even greater when it is accompanied by other abnormalities. Therefore, ultrasound and prenatal diagnosis are recommended. Pregnancy outcomes tend to be less favorable when NT thickening is accompanied by additional abnormalities, and a progressive increase in NT thickness is generally associated with a declining prognosis.

## 1. Introduction

Nuchal translucency (NT) refers to the subcutaneous fluid that is visualized behind the fetal neck on ultrasound during the first trimester. NT is a powerful indicator of aneuploidies when measured in the first trimester, which is when the crown–rump length (CRL) is measured at 45–84 mm, typically at gestational weeks 11^+0^ to 13^+6^ [1]. A thicker NT corresponds to a greater likelihood of chromosomal and structural abnormalities [2]. The normal NT range changes with increasing gestational age; however, there is controversy regarding the use of NT thickness in prenatal diagnosis. Some scholars have suggested defining NT thickening as 3.5 mm [3] or using a gestation‐specific threshold of ≥ 99^th^percentile (P_99_) [4] whereas others have suggested lower thresholds, such as 3.0 mm [5, 6] or the P_95_ [7, 8], for offering genetic counseling and prenatal diagnosis. The International Society for Ultrasound in Obstetrics and Gynecology does not specify a definition of “increased NT”; however, the American College of Obstetricians and Gynecologists and the Society for Maternal Fetal Medicine now recommend thresholds of 3.0 mm or the 99^th^ percentile. At present, the use of obstetric ultrasound in China generally follows the measurement criteria for NT according to the British Fetal Medicine Foundation. However, a corresponding normal NT range that is suitable for local pregnant women is lacking; moreover, in recent years, doubts have been raised regarding the use of an NT thickness ≥ 3.0 mm as the screening threshold. Currently, controversy exists with respect to whether NT thickening affects pregnancy outcomes in fetuses. Some studies have demonstrated that when fetal structural and chromosomal abnormalities are excluded, there is no significant difference in the incidence of adverse pregnancy outcomes compared with that in the general population [9] however, other studies have reported that fetuses still exhibit high risks of poor outcomes even when genetic analysis indicates a normal result [10] Therefore, the present study is aimed at exploring the normal range of NT measurements for fetuses at 11–13^+6^ weeks of gestation in Beijing, along with evaluating the prenatal diagnosis results and pregnancy outcomes associated with different NT thicknesses.

## 2. Materials and Methods

This retrospective study involved 239,276 NT measurements performed in Beijing between January 2022 and December 2023. NT screening is a routine screening program for early pregnancy in Beijing. Prior to screening, informed consent and follow‐up data were obtained and recorded in the Beijing Maternal and Child Phase III System.

A total of 3328 pregnant women with fetal NT thickness ≥ P_95_ were included in this study. The P_95_ and P_99_ of NT thickness were defined according to the normal reference ranges for NT in early pregnancy established by Wang Shuang et al. [8] in Beijing; these ranges were used as the cutoff values for increased NT. During data review, other morphological anomalies, such as fetal hydrops, congenital heart disease (CHD), and nasal bone abnormalities, were also detected during early‐ and/or mid‐pregnancy ultrasound screening. Prenatal diagnosis outcomes, including 1474 patients with an ultrasound diagnosis, 1473 patients with an invasive diagnosis, 865 patients with NIPT results, and pregnancy outcomes of all patients, were investigated. Ultrasound diagnosis was performed by senior professional physicians with ultrasound diagnostic qualifications from prenatal diagnostic institutions. The diagnosis was completed within 3 days after referral by screening institutions to ensure consistency in evaluating NT thickness. Invasive prenatal diagnosis was performed via chorionic villus sampling (CVS) or amniocentesis (AC) after informed consent was obtained from the patients, followed by karyotyping and chromosomal microarray analysis (CMA) or copy number variation (CNV) testing. All patients were assigned to one of two groups according to whether they had other concomitant abnormalities irrespective of gestational age at diagnosis (Groups A and B), and patients within the groups were divided into five subgroups (a–e) based on NT thickness. Subgroup a included patients with P_95_ ≤ NT < 3.0 mm, Subgroup b included patients with 3.0 mm ≤ NT < 4.0 mm, Subgroup c included patients with 4.0 mm ≤ NT < 5.0 mm, Subgroup d included patients with 5.0 mm ≤ NT < 6.0 mm, and Subgroup e included patients with NT ≥ 6.0 mm. Data on all first‐ and second‐trimester ultrasound screenings, noninvasive prenatal testing (NIPT), chromosomal and genetic testing results, and pregnancy outcomes during the study period were obtained from the Beijing Maternal and Child Phase III Information System database. Data on pregnancy outcomes were obtained from hospital records and through telephone interviews. Babies delivered in hospitals in Beijing were followed up via a delivery record form, which was saved in the information system. Pregnant women who gave birth outside a Beijing midwifery institution or who terminated their pregnancy were followed up to obtain data on fetal outcomes via telephone, and the data were recorded in the information system.

It is imperative to underscore the notion that the current protocol for prenatal screening and diagnostic services in Beijing explicitly stipulates that when the NT thickness is ≥ P_95_, prenatal diagnostic evaluation is strongly recommended. However, if the pregnant individual declines invasive diagnostic procedures, NIPT may be offered as an alternative screening option, which is contingent upon informed consent and shared decision‐making between the healthcare provider and the patient. Notably, in this study, instances where NIPT was performed without implementing the standardized combined screening protocol were observed.

The IBM SPSS Statistics (Version 29.0) statistical program was used for the statistical analysis. Quantitative data with a normal distribution are expressed as the means and standard deviations. A *t*‐test or Mann–Whitney *U* test was used to compare continuous data. Additionally, comparisons of categorical data were performed via Pearson′s chi‐square (*χ* ^2^) test. For subgroups with small sample sizes (expected cell count < 5), Fisher′s exact test was applied. Given the ordered nature of the subgroups (a–e), Cochran–Armitage trend tests were performed to assess monotonic trends. Binary logistic regression analysis was performed to evaluate the associations between NT thickness, pregnancy outcomes, and other factors. The results are reported as odds ratios (ORs) with 95% confidence intervals (CIs). A *p* value less than 0.05 was considered to indicate statistical significance.

## 3. Results

### 3.1. General Information

**Figure 1:**
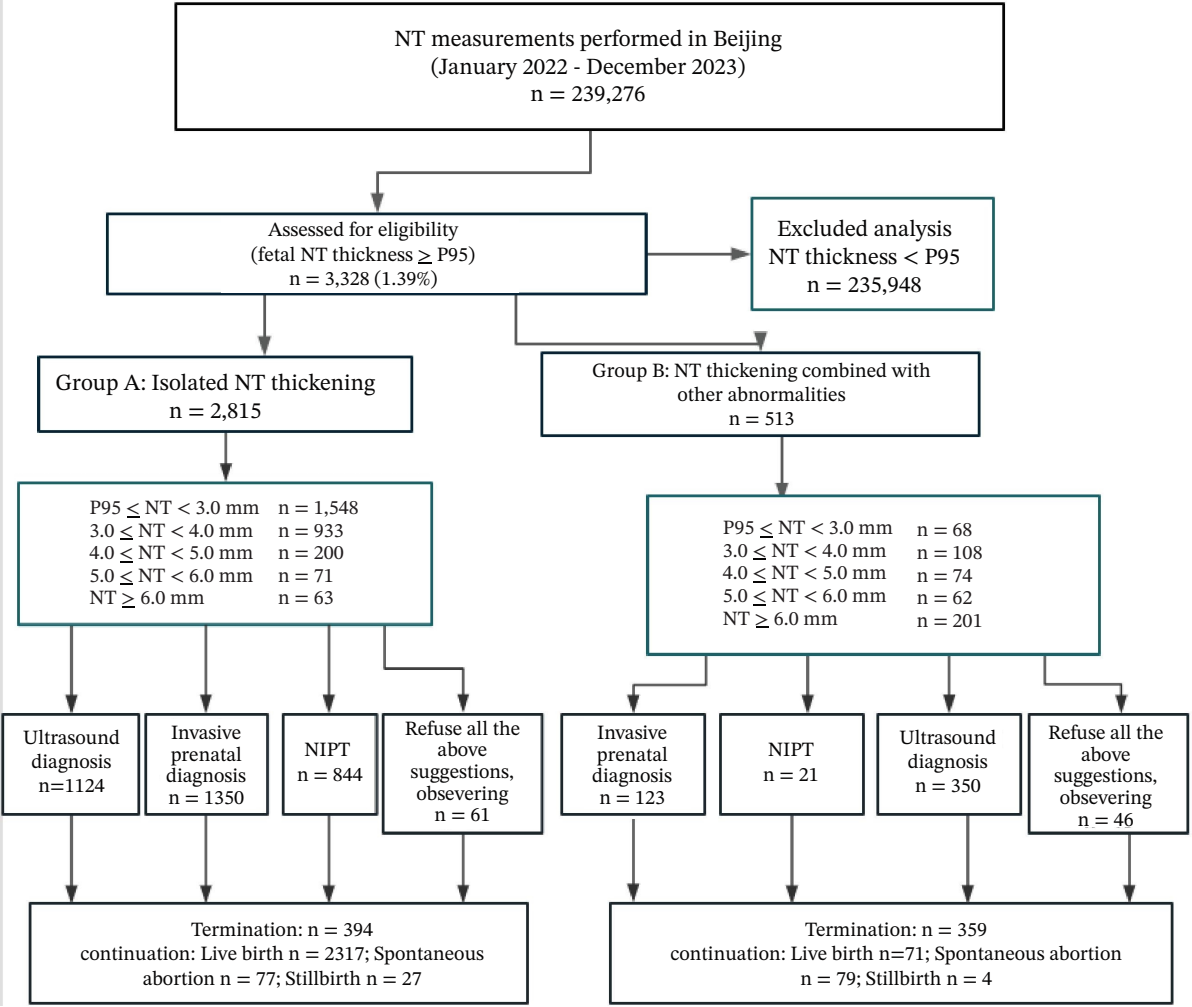
Flow diagram of participant selection.

NT scans were performed on 239,276 pregnancies between January 2022 and December 2023. Among them, 3328 patients had NT values above the P_95_ (1.39%, 3,328/239,276), 2815 patients (84.59%) demonstrated isolated NT thickening (Group A), and 513 patients (15.41%) demonstrated NT thickening combined with other abnormalities detected at any gestational age (Group B). The participant selection process is illustrated in Figure 1.

The details are presented in Table 1. The mean age (32.6 ± 4.61 years) and mean NT thickness (5.65 ± 2.76 mm) were greater in Group B than in Group A (32.18 ± 4.17 years and 3.17 ± 0.94 mm, respectively), and the difference was significant for both characteristics (*t* = 19.15, *p* < 0.001 and Z = 25.71, *p* < 0.001, respectively).

**Table 1:** Grouping details of the 3328 patients with NT thickening.

Different NT thicknesses	Total (*n* = 3328)	Group A (*n* = 2815)	Group B (*n* = 513)
P_95_ ≤ NT < 3.0 mm	1616	1548	68
3.0 mm ≤ NT < 4.0 mm	1041	933	108
4.0 mm ≤ NT < 5.0 mm	274	200	74
5.0 mm ≤ NT < 6.0 mm	133	71	62
NT ≥ 6.0 mm	264	63	201

### 3.2. Analysis of Prenatal Diagnosis and Screening

Among the 3328 patients, 44.29% (1474/3328) underwent prenatal ultrasound diagnosis, with a main diagnostic concordance rate of 83.24% (1227/1474) between ultrasound screening and diagnosis. Among these patients, 1124 underwent ultrasound diagnosis in Group A, with a concordance rate of 78.47% (882/1124). Moreover, 350 patients underwent ultrasound diagnosis in Group B, with a concordance rate of 98.57% (345/350). The concordance rate increased with increasing NT thickness and was significant for both Groups A and B (*χ* ^2^ = 43.28, *p* < 0.001 vs. *χ* ^2^ = 14.20, *p* = 0.007, respectively). When the NT thickness was less than 4 mm and the same NT thickness range was used, the concordance rate in Group B was greater than that in Group A and was consistent with the overall accuracy *χ* ^2^ = 77.31 (*p* < 0.001).

Overall, 44.26% (1473/3328) of the patients underwent invasive prenatal diagnosis; among them, 333 had chromosomal abnormalities, thus leading to a chromosomal abnormality rate of 22.61%. With respect to P_95_ ≤ NT < 3.0 mm, the incidence of chromosomal abnormalities in Groups A and B was 13.66% and 23.08%, respectively. When the NT thickness was greater than 6.0 mm, the incidence of chromosomal abnormalities in Groups A and B was 53.13% and 66.67%, respectively, indicating that the incidence of chromosomal abnormalities was greater in Group B than in Group A (*χ* ^2^ = 62.80, *p* < 0.001). The chromosomal abnormality rate increased with increasing NT thickening in Group A (Z = 7.61, *p* < 0.001) and Group B (*Ζ* = 3.32, *p* < 0.001) (Table 2). NT thickening cutoff values of P_95_ or 3.0 mm resulted in significantly different rates of abnormal chromosome detection, with rates of 20.00% (270/1350) and 26.05% (180/691), respectively (*χ* ^2^ = 9.48, *p* = 0.002), in Group A, along with nonsignificant differences of 51.22% (63/123) and 58.76% (57/97), respectively (*χ* ^2^ = 1.09, *p* = 0.30), in Group B (Table 2).

**Table 2:** Chromosome results with different NT thicknesses.

NT thickness	Group A			Group B			*χ* ^2^	*p*
	Total	Normal	Abnormal/*n*(%)	Total	Normal	Abnormal/*n*(%)		
P_95_ ≤ NT < 3.0	659	569	90 (13.7)	26	20	6 (23.1)	1.84	0.18
3.0 mm ≤ NT < 4.0 mm	514	398	116 (22.6)	33	16	17 (51.5)	14.12	< 0.001
4.0 mm ≤ NT < 5.0 mm	123	87	36 (29.27)	21	11	10 (47.6)	2.78	0.10
5.0 mm ≤ NT < 6.0 mm	22	11	11 (50.0)	7	1	6 (85.71)	2.14	0.14
NT ≥ 6.0 mm	32	15	17 (53.1)	36	12	24 (66.7)	1.30	0.26
Z	—	—	7.61	—	—	3.32	—	—
*p* value	—	—	<0.001	—	—	< 0.001	—	—
Total	1350	1080	270 (20.0)	123	60	63 (51.2)	62.80	< 0.001

The most common chromosomal abnormality was Trisomy 21, which was present in 46.55% of all patients (155/333), followed by sex chromosome abnormalities (12.24%, 41/333) and Trisomy 18 (9.31%, 31/333). The incidence of Trisomy 21 in Groups A and B was 9.11% and 27.64%, respectively, and the difference between the groups was significant (*χ*2 = 35.45, *p* < 0.001). Significant differences were also observed in the rates of Trisomy 18 (1.33% vs. 10.57%, *χ* ^2^ = 47.71, *p* < 0.001), Trisomy 13 (0.37% vs. 2.44%, *χ* ^2^ = 9.14, *p* = 0.003), and sex chromosome abnormalities (2.44% vs. 6.69%, *χ* ^2^ = 7.12, *p* = 0.008). With respect to P_95_ ≤ NT < 3.0 mm, the incidence of Trisomy 21 was 5.92% (39/659) and 11.54% (3/26) in Groups A and B, respectively.

In addition, 865 pregnant women underwent NIPT screening, which revealed 23 fetuses with a high risk of Trisomy 21, 4 fetuses with a high risk of Trisomy 18, 2 fetuses with a high risk of Trisomy 13, 5 fetuses with sex chromosome abnormalities and 5 fetuses with other chromosome abnormalities. The prenatal diagnosis rate of fetuses at high risk for trisomies 21/18/13 was 79.31% (23/29), and all of the diagnosed fetuses were confirmed to be abnormal. Among those who declined prenatal diagnosis, there was one case of spontaneous abortion and five cases of elective pregnancy termination.

Ultrasound screening revealed NT thickness combined with other abnormalities, including cystic hygroma, fetal hydrops, CHD, and abnormal nasal bone development. Herein, regardless of gestational age, the most common structural abnormalities and soft indicators included CHD (21.25%, 109/513) and cystic hygroma (40.35%, 207/513) (Table 3).

**Table 3:** Other ultrasound abnormalities according to NT thickness.

NT thickness	Congenital heart disease	Abnormal organs other than heart	Nasal bone abnormalities	Other bone abnormalities	Fetal hydrops	Cystic hygroma	Other abnormalities
P_95_ ≤ NT < 3.0 mm	17	17	12	9	4	11	7
3.0 mm ≤ NT < 4.0 mm	20	38	22	9	16	22	13
4.0 mm ≤ NT < 5.0 mm	15	17	15	7	30	25	5
5.0 mm ≤ NT < 6.0 mm	10	14	3	4	18	35	3
NT ≥ 6.0 mm	47	47	17	20	89	114	12
Total	109	133	69	49	157	207	40

In this study, among fetuses with an NT thickness ≥ P_99_, 103 had CHD, accounting for 4.25% of the fetuses (103/2425). Moreover, among fetuses with NT < P_99_, 6 had CHD, accounting for 0.66% of the fetuses (6/903). Statistical analysis of the relationship between NT thickness and CHD revealed a *χ* ^2^ value of 26.66 and an OR of 6.63 according to univariate logistic regression analysis, with a 95% CI of 2.90–15.16 (*p* < 0.001). Multivariate logistic regression analysis (adjusted for maternal age, gravidity, and parity) revealed an OR of 6.65, with a 95% CI of 2.91–15.21 (*p* < 0.001) (Table 4).

**Table 4:** Relationships among age, number of pregnancies, number of deliveries, NT thickness, and CHD.

Variables	Univariate		Multivariate	
	OR (95% CI)	*p*	OR (95% CI)	*p*
Age	1.02 (0.97–1.07)	0.42	0.97 (0.93–1.02)	0.282
Number of pregnancies	0.91 (0.75–1.09)	0.29	1.28 (0.98–1.69)	0.075
Number of deliveries	1.01 (0.72–1.42)	0.94	0.76 (0.47–1.24)	0.276
NT thickness^a^	6.63 (2.90–15.16)	< 0.001	6.65 (2.91–15.21)	< 0.001

^a^NT thickness is demarcated, with a value of ≥P_99_ functioning as the dividing line.

### 3.3. Pregnancy Outcomes of Patients With Increased NT Thickness

Among all pregnancies with NT thickening, the overall live birth rate was 71.75% (2388/3328), with a rate of 69.62% (2317/3328) in Group A and a rate of 2.13% (71/3328) in Group B. When analyzed separately, the live birth rate was 82.31% (2317/2815) in Group A and 13.84% (71/513) in Group B, with a highly significant difference between the groups (*χ*2 = 1003.70, *p* < 0.001). Moreover, the rates of spontaneous abortion/embryo arrest were 2.74% in Group A and 15.40% in Group B (*χ*2 = 155.78, *p* < 0.001). The percentage of miscarriages or stillbirths among patients with increased NT and associated malformations was 53.90% (83/154). The rates of elective termination of pregnancy (14.00% vs. 70.00%, *χ*2 = 776.85; *p* < 0.001) were also significantly different (Table 5).

**Table 5:** Pregnancy outcomes in Groups A and B.

Pregnancy outcomes	Total (*n* = 3328)	Group A (*n* = 2815)	Group B (*n* = 513)	*χ* ^2^	*p*
Live birth, *n* (%)	2388 (71.8)	2317 (82.3)	71 (13.8)	1003.70	< 0.001
Stillbirth, *n* (%)	31 (0.9)	27 (1.0)	4 (0.8)	0.15	0.70
Embryo arrest/spontaneous abortion, *n* (%)	156 (4.7)	77 (2.7)	79 (15.4)	155.78	< 0.001
Termination of pregnancy, *n* (%)	753 (22.6)	394 (14.00)	359 (70.0)	776.85	< 0.001

After excluding the subgroup that elected pregnancy termination, pregnancy outcomes were analyzed among those who chose to continue gestation under surveillance. In Group A, statistically significant differences were detected among the different NT thickness subgroups in terms of live birth rates (*χ* ^2^ = 21.37, Z = −11.49, *p* < 0.001), embryo arrest or spontaneous abortion rates (*χ* ^2^ = 215.93, Z = 11.89, *p* < 0.001), and stillbirth rates (*χ* ^2^ = 13.36, Z = 2.31, *p* < 0.05)(Table 6). In Group B, statistically significant differences were observed only in the live birth rates (*χ* ^2^ = 28.64, Z = −5.33, *p* < 0.001) and embryo arrest or spontaneous abortion rates (*χ* ^2^ = 28.96, Z = 5.34, *p* < 0.001). Furthermore, no statistically significant difference in the stillbirth rate was identified among the subgroups within Group B (*χ* ^2^ = 1.16, Z = −0.07, *p* > 0.05) (Table 7).

**Table 6:** Pregnancy outcomes of continued pregnancy according to NT thickness in Groups A and B. S1: Pregnancy outcomes according to NT thickness in Group A.

Pregnancy outcomes	Group A (*n*, %)								
	a	b	c	d	e	*χ* ^2^	*p*	*z*	*p*
Live birth rate	1404 (97.57)	751 (95.91)	129 (89.58)	22 (68.75)	11 (47.82)	21.37	< 0.001	−11.49	< 0.001
Stillbirth rate	14 (0.97)	8 (1.02)	2 (1.39)	1 (3.12)	2 (8.70)	13.36	0.01	2.31	−0.02
Embryo arrest/spontaneous abortion rate	21 (1.46)	24 (3.07)	13 (9.03)	9 (28.13)	10 (47.48)	215.93	< 0.001	11.89	< 0.001
Total	1439 (100)	783 (100)	144 (100)	32 (100)	23 (100)	—	—	—	—

*Note:* a, P_95_ ≤ NT < 3.0 mm; b, 3.0 mm ≤ NT < 4.0 mm; c, 4.0 mm ≤ NT < 5.0 mm; d, 5.0 mm ≤ N < 6.0 mm; e, NT ≥ 6.0 mm.

**Table 7:** S2: Pregnancy outcomes according to NT thickness in Group B.

Pregnancy outcomes	Group B (*n*, %)								
	a	b	c	d	e	*χ* ^2^	*p*	*z*	*p*
Live birth rate	24 (75.00)	23 (62.16)	9 (42.86)	6 (31.58)	9 (20.00)	28.64	< 0.001	−5.33	< 0.001
Stillbirth rate	1 (3.12)	1 (2.70)	0 (0.00)	1 (5.26)	1 (2.22)	1.16	0.89	−0.07	0.95
Embryo arrest/spontaneous abortion rate	7 (21.88)	13 (35.14)	12 (57.14)	12 (63.16)	35 (77.78)	28.96	< 0.001	5.34	< 0.001
Total	32 (100)	37 (100)	21 (100)	19 (100)	45 (100)	—	—	—	—

*Note:* a, P_95_ ≤ NT < 3.0 mm; b, 3.0 mm ≤ NT < 4.0 mm; c, 4.0 mm ≤ NT<5.0 mm; d, 5.0 mm ≤ NT < 6.0 mm; e, NT ≥ 6.0 mm.

The reasons for elective termination of pregnancy included chromosomal abnormalities, early pregnancy ultrasound abnormalities, and mid‐pregnancy ultrasound abnormalities, among others. The rate of NT thickening combined with other abnormalities was 50.33% (379/753), and the rate of NT thickening associated with chromosomal abnormalities was 31.08% (234/753). Moreover, the rate of NT thickening associated with isolated NT thickening was 17.26% (130/753), and the rate of NT thickening associated with ultrasound abnormalities in mid‐pregnancy was 10.89% (82/753). The rate of NT thickening associated with other abnormalities was 1.33% (10/753) (Table 8).

**Table 8:** Reasons for terminating pregnancy.

Reasons	Group A					Group B				
	a	b	c	d	e	a	b	c	d	e
Combined other abnormalities in early pregnancy	0	0	0	0	0	31	56	42	38	129
Chromosomal abnormalities	51	78	25	11	17	5	14	9	4	21
Combined abnormalities in mid‐pregnancy	36	21	8	4	3	0	1	2	1	6
Isolated NT thickening	19	46	22	23	20	0	0	0	0	0
Other	3	5	1	1	0	0	0	0	0	0
Total	109	150	56	39	40	36	71	53	43	156

*Note:* a, P_95_ ≤ NT < 3.0 mm; b, 3.0 mm ≤ NT < 4.0 mm; c, 4.0 mm ≤ NT < 5.0 mm; d, 5.0 mm ≤ NT < 6.0 mm; e, NT ≥ 6.0 mm.

The rate of termination of pregnancy among those with isolated NT was significantly lower than that among those with combined abnormalities (14.00% (394/2815) versus 70.00% (359/513), *χ* ^2^ = 773.38, *p* < 0.001). The proportion of terminated pregnancies with isolated NT thickening increased with increasing NT thickness, regardless of the group (Group A, Z = 17.52, *p* < 0.001; Group B, Z = 3.77, *p* < 0.001) (Table 9).

**Table 9:** Number of pregnancy terminations in the different NT thickness groups.

NT thickness	Group A		Group B		*χ* ^2^	*p*
	Total	Termination/*n*(%)	Total	Termination/*n*(%)		
P_95_ ≤ NT < 3.0 mm	1548	109 (7.04)	68	36 (52.94)	158.06	< 0.001
3.0 mm ≤ NT < 4.0 mm	933	150 (16.08)	108	71 (65.74)	142.05	< 0.001
4.0 mm ≤ NT < 5.0 mm	200	56 (28.00)	74	53 (71.62)	43.05	< 0.001
5.0 mm ≤ NT < 6.0 mm	71	39 (54.93)	62	43 (69.35)	2.83	0.09
NT ≥ 6.0 mm	63	40 (63.49)	201	156 (77.61)	4.88	0.03
Total	2815	394 (14.00)	513	359 (70.00)	773.48	< 0.001
Z	—	17.52	—	3.77	—	—
*p* value	—	< 0.001	—	< 0.001	—	—

After women who underwent termination were excluded, ordered logistic regression was performed on three outcomes: live birth, stillbirth, and spontaneous abortion. For live birth, both age (univariate OR = 1.03, *p* = 0.008; multivariate OR = 1.03, *p* = 0.006) and NT thickness (univariate OR = 0.52, *p* < 0.001; multivariate OR = 0.53, *p* < 0.001) were significant predictors. For spontaneous abortion, only NT thickness remained significant (univariate OR = 2.35, *p* = 0.001; multivariate OR = 2.10, *p* = 0.005), whereas age was not (*p* = 0.62 and *p* = 0.85, respectively). For stillbirth, both age (univariate OR = 0.96, *p* = 0.008; multivariate OR = 0.98, *p* = 0.04) and NT thickness (univariate OR = 1.85, *p* < 0.001; multivariate OR = 1.65, *p* < 0.001) were significant (Table 10). These correlations were also observed in Group A (*p* < 0.001)(Table 11); however, maternal age was not correlated with live birth or termination or stillbirth in Group B (Table 12).

**Table 10:** Relationships among age, NT thickness, and pregnancy outcomes among all patients.

Variables	Live births among all				Spontaneous abortions among all				Stillbirths among all			
	Univariate		Multivariate		Univariate		Multivariate		Univariate		Multivariate	
	OR (95% CI)	*p*	OR (95% CI)	*p*	OR (95% CI)	*p*	OR (95% CI)	*p*	OR (95% CI)	*p*	OR (95% CI)	*p*
Age	1.03 (1.01–1.06)	0.008	1.03 (1.01–1.06)	0.006	1.02 (0.94–1.11)	0.62	1.01 (0.92–1.10)	0.85	0.96 (0.93–0.99)	0.008	0.98 (0.95–1.00)	0.04
NT Thickness	0.52 (0.38–0.71)	< 0.001	0.53 (0.38–0.73)	< 0.001	2.35 (1.45–3.80)	0.001	2.10 (1.25–3.52)	0.005	1.85 (1.48–2.30)	< 0.001	1.65 (1.35–2.02)	< 0.001

**Table 11:** S2: Relationships among age, NT thickness, and pregnancy outcomes in Group A patients.

Variables	Live births in Group A				Spontaneous abortions in Group A				Stillbirths in Group A			
	Univariate		Multivariate		Univariate		Multivariate		Univariate		Multivariate	
	OR (95% CI)	*p*	OR (95% CI)	*p*	OR (95% CI)	*p*	OR (95% CI)	*p*	OR (95% CI)	*p*	OR (95% CI)	*p*
Age	1.03 (1.01–1.05)	< 0.001	1.03 (1.01–1.05)	< 0.001	0.97 (0.93–1.03)	0.48	0.98 (0.94–1.04)	0.58	0.97 (0.95–0.99)	< 0.001	0.98 (0.96–1.00)	0.002
NT Thickness	0.28 (0.24–0.33)	< 0.001	0.31 (0.26–0.37)	< 0.001	2.30 (1.25–4.22)	0.008	2.15 (1.15–4.02)	0.015	2.60 (2.12–3.18)	< 0.001	2.40 (1.93–2.98)	< 0.001

**Table 12:** S3: Relationships among age, NT thickness, and pregnancy outcomes in Group B patients.

Variables	Live births in Group B				Spontaneous abortions in Group B				Stillbirths in Group B			
	Univariate		Multivariate		Univariate		Multivariate		Univariate		Multivariate	
	OR (95% CI)	*p*	OR (95% CI)	*p*	OR (95% CI)	*p*	OR (95% CI)	*p*	OR (95% CI)	*p*	OR (95% CI)	*p*
Age	1.01 (0.98–1.04)	0.52	1.00 (0.97–1.03)	0.85	0.99 (0.92–1.07)	0.82	0.98 (0.90–1.06)	0.63	1.00 (0.97–1.03)	0.91	0.99 (0.96–1.02)	0.48
NT Thickness	0.42 (0.33–0.54)	< 0.001	0.45 (0.35–0.58)	< 0.001	2.45 (1.20–5.00)	0.014	2.20 (1.05–4.60)	0.036	1.68 (1.35–2.09)	< 0.001	1.60 (1.28–2.00)	< 0.001

## 4. Discussion

With the rapid development of prenatal diagnostic technology, the use of new technologies for prenatal screening and diagnosis has become important for reducing the incidence of birth defects. In the 1990s, NT thickening was demonstrated to be an important indicator for screening fetal chromosomal abnormalities during early pregnancy. In this study, we focused on prenatal diagnosis and the outcomes of pregnancies with an NT thickness ≥ P_95_. The present study included 3328 patients, the majority of whom had isolated NT thickening (84.59% of the included patients). Notably, 1616 patients had P_95_ ≤ NT < 3.0 mm. In Beijing, when prenatal ultrasound screening indicates NT thickening, the patient should be referred to a diagnostic institution for an ultrasound diagnosis. If the ultrasound diagnosis again indicates NT thickening, invasive prenatal diagnosis is recommended. In this study, 44.29% of the patients received an ultrasound‐based diagnosis. With respect to the diagnostic concordance rate for ultrasound, a greater NT indicates greater concordance. The concordance rate for nonisolated NT thickening was greater than that for isolated NT thickening, with an average concordance rate of 83.24%; moreover, isolated NT thickening of less than 3.0 mm exhibited the worst concordance rate. In summary, physicians performing prenatal ultrasound screening in Beijing generally demonstrate a high concordance rate in measuring NT; however, training is still needed for measurements of isolated NT thickening and NT thicknesses of less than 3.0 mm. Moreover, pregnant women with an NT thickness measuring less than 3.0 mm at screening institutions should be further evaluated to avoid miscarriage caused by measurement errors.

In our study, 44.26% of the patients underwent invasive prenatal diagnosis, with a chromosomal abnormality rate of 22.61%. These results are similar to those of previous research [11], and the rate increased with increasing NT thickness. The use of different NT thickening cutoff values resulted in different abnormal chromosome number detection rates. In this study, the reduction rate of chromosomal abnormalities when an NT of 3.0 mm was used as the cutoff value was greater than that when it was used as the cutoff value for P_95_; however, 10% of chromosomal abnormalities still occurred between the P_95_ and 3.0 mm values. These results suggest that when the NT thickness is ≥ P_95_, invasive prenatal diagnosis should be recommended. The most common chromosomal abnormality associated with NT thickening was Trisomy 21, which is consistent with the findings of previous studies [12]. We also reported that the rate of chromosomal abnormalities in fetuses with nonisolated NT thickening was greater than that in fetuses with isolated NT thickening. Nicolaids et al. [13] confirmed that the finding of multiple abnormalities in fetuses on ultrasound is strongly correlated with chromosomal abnormalities. More than 60% of fetuses with chromosomal abnormalities demonstrated multiple abnormalities on ultrasound during pregnancy, which is consistent with the findings of a previous study [14]. NT thickening is only a soft ultrasound indicator, which could explain the lower incidence of chromosomal abnormalities observed in fetuses with isolated NT thickening than in those with nonisolated NT thickening. Therefore, in fetuses with NT thickening combined with other abnormalities, the possibility of chromosomal abnormalities should be considered.

In our study, when NT thickening was combined with structural abnormalities, the most common structural abnormality was CHD, which is consistent with the literature. Currently, the relationship between NT thickening and CHD is the most studied relationship. Previous studies have suggested that NT thickness has a certain value in predicting the occurrence of CHD [15], whereas other studies have reported that an NT thickness ≥ P_95_ is correlated with increased mortality in children with simple CHD [16]. Other studies have reported strong associations between CHD and increased NT thickness [17], as well as correlations between NT thickening and genetic alterations in heart defects [18]. In our research, the incidence of heart defects was greater than that of other structural abnormalities, thus indicating a relationship between NT thickening and heart defects. In particular, when the NT thickness was ≥ P_99_, the incidence rate of CHD significantly increased, which is consistent with the findings of previous studies [19]. Moreover, previous studies have suggested that septations could be visualized in all fetuses with increased NT thickness when an appropriate gain in the transverse plane is used by the sonographer [20]; furthermore, the authors proposed that cystic hygroma does not constitute a distinct entity in the first trimester, conferring a special risk status independent of NT thickness. However, other studies have revealed that the presence of septations tends to be worse in fetuses with nonisolated NT thickening than in fetuses with isolated NT thickening [21]. In our study, cystic hygroma was observed in 207 patients. In addition to cystic hygroma, more than half of the cases were associated with other structural malformations, including heart and skeletal malformations, which is consistent with the literature [11, 12]. The identification of cystic hygroma as a special risk factor independent of NT thickness is difficult. More individual cases may be needed in the future to analyze whether cystic hygroma is an independent risk factor.

NT thickening in early pregnancy is a soft indicator for predicting adverse fetal outcomes; moreover, in a previous study [22], the probability of structural abnormalities was greater in fetuses with NT thickening than in the general population. In our study, 82.31% of patients with isolated fetal NT thickening had normal pregnancy outcomes, whereas 13.84% of patients with other fetal abnormalities had normal pregnancy outcomes. The difference between the two groups was significant, which is consistent with the study by Bilardo et al. [23]. Moreover, within the same NT thickness range, the live birth rate among the fetuses with isolated NT thickening was significantly greater than that among the fetuses with other abnormalities. Furthermore, when women whose pregnancies were terminated were excluded, the live birth rate of the fetuses decreased, and the stillbirth rate and embryo arrest/spontaneous abortion rate increased with increasing NT thickness regardless of whether additional fetal anomalies were present. However, the stillbirth subgroup was very small; therefore, these findings should be interpreted with caution. In addition, the rate of pregnancy termination among pregnant women with isolated NT thickening was significantly lower than that among pregnant women with other abnormalities, and the termination rate increased with increasing NT thickening, which was similar to the natural miscarriage rate. These results are similar to those of Maymon et al. [24]. However, the results are not entirely similar to those of Piazze et al. [25], who reported that an increase in NT thickness is significantly associated with the occurrence of preterm delivery. However, Piazze et al. did not report a significant relationship between an increase in NT and abortion threat. In this study, the main reasons for pregnancy termination included other abnormalities and chromosomal abnormalities, which accounted for 81.41% of the terminated pregnancies. Additionally, 17.26% of the patients decided to end their pregnancy because of NT thickening, with NT thicknesses between 3.0 mm and 4.0 mm demonstrating the highest termination rate of 6.11%, and other NT thicknesses exhibited a termination rate of 2.52%–3.05%. With respect to the live birth rate, pregnant women who choose to terminate their pregnancies should undergo invasive prenatal diagnosis to exclude chromosomal abnormalities. If the chromosomes are normal, midterm ultrasound screening and cardiac ultrasound examination should be performed. In addition, these findings suggest that clinical physicians should increase their knowledge of NT thickening to provide better advice to pregnant women.

The present study had several limitations. First, not all pregnant women with NT thickness were followed through to their actual pregnancy outcomes, as a proportion of women elected to terminate their pregnancies for various personal reasons. This selective attrition introduces potential verification bias: The observed pregnancy outcomes represent only those who continued their pregnancies, and the characteristics of women who chose termination may differ systematically from those who did not. Second, invasive prenatal diagnosis was not performed universally. All prenatal screening and diagnostic procedures were conducted on a strictly voluntary basis in accordance with national guidelines and institutional policies. This means that chromosomal analysis was available only for women who consented to invasive testing, creating significant selection bias. Women who opted for invasive diagnosis may have had higher baseline risk profiles (e.g., a thicker NT, advanced maternal age, or abnormal serum markers), which could inflate the apparent association between NT thickness and adverse outcomes. Third, because successful birth of the fetus indicates a normal pregnancy outcome, some genetic syndromes and neurodevelopmental disorders may have been overlooked. However, in a large study involving more than 220,000 fetuses followed up to the age of 4 years, few children in the NT thickness ≥ P_99_ group were diagnosed with intellectual disability. Moreover, cerebral palsy, epilepsy, and febrile seizures are not associated with an NT thickness ≥ 99th percentile, and the risk of postnatal developmental delay in fetuses with isolated NT thickening is not significantly different from that in the general population [10, 26, 27]. Despite these limitations, the study findings remain clinically meaningful. The strong and independent association between NT thickness and adverse pregnancy outcomes—observed even after adjusting for maternal age—supports the continued use of NT thickness as a first‐trimester risk stratification measurement tool. In real‐world clinical practice, where not all women undergo invasive testing, NT thickness provides a noninvasive, early, and accessible marker that can guide counseling, inform decision‐making, and identify high‐risk pregnancies that warrant closer surveillance. Although the absolute risk estimates may be affected by selection bias, the relative ranking of risk across NT strata is likely preserved, making NT a valuable tool for clinical triage even in settings with incomplete diagnostic follow‐up.

## 5. Conclusions

In summary, fetuses with a thickened NT are more likely to develop structural and chromosomal abnormalities. Moreover, patients with an isolated fetal NT thickness ≥ P_95_ appear to have relatively favorable pregnancy outcomes. In contrast, among those with other fetal abnormalities, the risks of adverse pregnancy outcomes, including miscarriage, termination of pregnancy, and spontaneous preterm birth, tend to increase. The results of the present study may offer useful reference information to support clinical counseling regarding the potential risks associated with NT thickness. On the basis of this study, further analysis of prospective studies with complete follow‐up outcomes, standardized diagnostic protocols, and long‐term neurodevelopmental assessments is warranted to validate and extend these findings to devise more effective pregnancy management strategies.

## Author Contributions

Yanchun Zhang designed the study, analyzed the data, and wrote the manuscript. Hongyan Xu sorted and reviewed the NT thickness database. Lijing Wang was responsible for reviewing the statistical data. Wen Zhang collected and analyzed the birth defect information and pregnancy outcomes. Kaibo Liu designed the study, guided the writing of the article and edited the manuscript. All the authors contributed to the article and approved the submitted version.

## Funding

This study was supported by the National Key Research and Development Program of China, 10.13039/501100012166, 2018YFC1002304; Beijing Municipal Health Commission High level Public Health Talent Project, 2022‐2‐028.

## Ethics Statement

This study was reviewed and approved by the Ethics Committee of Beijing Obstetrics and Gynecology Hospital, Capital Medical University (No. 2019‐KY‐025‐01). The approval covers both the primary study and subsequent analyses of the related data. Written informed consent was obtained from all participants.

## Consent

The collected data were routinely obtained during standard care, and all the patients signed informed consent forms for prenatal screening and diagnosis. Patients or their legal guardians were informed that these data may be used in studies conducted within the services.

## Conflicts of Interest

The authors declare no conflicts of interest.

## Data Availability

The data that support the findings of this study are available upon request from the corresponding author.
